# A new aspect of chronic pain as a lifestyle-related disease

**DOI:** 10.1016/j.ynpai.2017.04.003

**Published:** 2017-04-17

**Authors:** Emiko Senba, Katsuya Kami

**Affiliations:** a Department of Physical Therapy, Osaka Yukioka College of Health Science, 1-1-41 Sojiji, Ibaraki-City, Osaka 567-0801, Japan; b Department of Rehabilitation Medicine, Wakayama Medical University, 811-1 Kimiidera, Wakayama City, Wakayama 641-8509, Japan

**Keywords:** CBP, chronic low back pain, DA, dopamine, delta FosB, delta FBJ murine osteosarcoma viral, FM, fibromyalgia, GABA, gamma-aminobutyric acid, HDAC, histone deacetylase, LDT, laterodorsal tegmental nucleus, LH, lateral hypothalamus, LHb, lateral habenula, mPFC, medial prefrontal cortex, NAc, nucleus accumbens, NPP, neuropathic pain, pCREB, phosphorylated cyclic AMP response element-binding protein, PPTg, pedunculopontine tegmental nucleus, PSL, partial sciatic nerve ligation, RMTg, rostromedial tegmental nucleus, TH, tyrosine hydroxylase, TMD, temporomandibular disorder, VTA, ventral tegmental area, VWR, voluntary wheel running, Exercise-induced hypoalgesia, Chronic pain, Ventral tegmental area, Dopamine, Laterodorsal tegmental nucleus, Physical activity/inactivity

## Abstract

Physical exercise has been established as a low-cost, safe, and effective way to manage chronic intractable pain. We investigated the underlying mechanisms of exercise-induced hypoalgesia (EIH) using a mouse model of neuropathic pain (NPP). Epigenetic changes in activated microglia and maintained GABA synthesis in the spinal dorsal horn may contribute to EIH. Voluntary exercise (VE), a strong reward for animals, also induced EIH, which may be due in part to the activation of dopamine (DA) neurons in the ventral tegmental area (VTA). VE increases the expression of pCREB in dopaminergic neurons in the VTA, which would enhance dopamine production, and thereby contributes to the activation of the mesolimbic reward system in NPP model mice. We demonstrated that neurons in the laterodorsal tegmental and pedunculopontine tegmental nuclei, a major input source of rewarding stimuli to the VTA, were activated by exercise.

Chronic pain is at least partly attributed to sedentary and inactive lifestyle as indicated by the Fear-avoidance model. Therefore, chronic pain could be recognized as a lifestyle-related disease. Physical activity/inactivity may be determined by genetic/epigenetic and neural factors encoded in our brain. The hypothalamus and reward system is closely related in the axis of food intake, energy metabolism and physical activity. Understanding the interactions between the mesolimbic DA system and the hypothalamus that sense and regulate energy balance is thus of significant importance. For example, proopiomelanocortin neurons and melanocortin 4 receptors may play a role in connecting these two systems. Therefore, in a certain sense, chronic pain and obesity may share common behavioral and neural pathology, i.e. physical inactivity, as a result of inactivation of the mesolimbic DA system. Exercise and increasing physical activity in daily life may be important in treating and preventing chronic pain, a life-style related disease.

## Highlights

• Activation of mesolimbic dopamine system underlies exercise-induced hypoalgesia.
• Interaction between mesolimbic system and hypothalamus determines physical activity.
• Changing the lifestyle inactive to active may attenuate and prevent chronic pain.

## Graphical abstract

**Figure:**
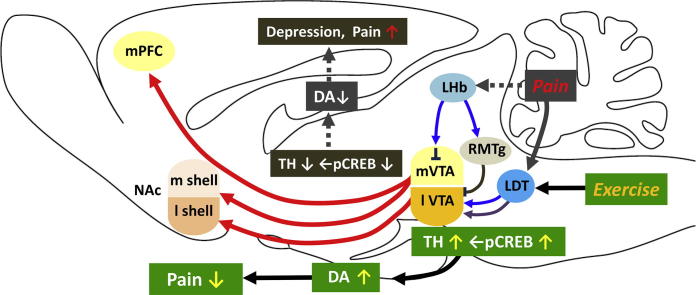

## Introduction

Neuropathic pain (NPP) is an intractable form of chronic pain that is produced by damage to and pressure on the peripheral and central nervous systems, and is the most difficult type of pain to treat among chronic pain diseases (Almeida et al., 2015, Jain, 2008). Pharmacological management of NPP has been challenged by clinicians with insufficient outcome. Since only 10–25% of patients respond to the first choice drugs for NPP, according to NNT (=number needed to treat) of these drugs (Finnerup et al., 2015), chronic pain patients suffer various side effects of having overdoses of these drugs for long period. On the other hand, non-pharmacological patient-oriented approaches have been proven to significantly attenuate chronic pain. One of those approaches is physical exercise, such as running or swimming. Relevant studies demonstrated that physical exercise in NPP model animals can significantly improve pain-related behaviors, such as mechanical allodynia and heat hyperalgesia (exercise-induced hypoalgesia: EIH) (Kuphal et al., 2007, Shen et al., 2013). However, the underlying mechanisms of how exercise attenuates NPP are not yet well understood. In addition, it is known that physical exercise in clinical patients attenuates their pain symptoms as well, and can appreciably improve their activities of daily living (ADL) (Ambrose and Golightly, 2015, Koltyn et al., 2014). However, exercise therapy is still not actively encouraged to patients with chronic pain because of the uncertainty of the mechanisms underlying EIH. Therefore, understanding these mechanisms will allow a compelling argument to be made for exercise therapy with the goal of improving chronic pain.

Emerging evidence from animal studies has identified several factors that work at different levels of the nervous system as playing critical roles to produce EIH in NPP model animals (Almeida et al., 2015, Bobinski et al., 2015, Chen et al., 2012, Cobianchi et al., 2010, Cobianchi et al., 2013, Kami et al., 2016a, Kami et al., 2016b, López-Álvarez et al., 2015, Shankarappa et al., 2011, Stagg et al., 2011). A line of research demonstrated that EIH is a hypoalgesia composed of multiple events including marked alterations in inflammatory cytokines, neurotrophins, neurotransmitters, endogenous opioids and histone acetylation in injured peripheral nerves, DRG and spinal dorsal horns in NPP model animals following physical exercise (For review, see Kami et al., 2017). In this review, we introduce our recent findings associated with EIH in NPP model animals and provide a new aspect of chronic pain as a lifestyle-related disease, and then discuss a future direction toward its therapeutic strategy.

## Exercise-induced changes in the spinal cord of NPP animals

A line of evidence supports the notion that glial cells in the spinal dorsal horn are key players in the pathogenetic process of NPP (Mika et al., 2013). In line with this notion, some studies showed that treadmill running and swimming in NPP model animals can significantly reduce the expression levels of CD11b, Iba-1 and glial fibrillary acidic protein (GFAP), which are reliable markers for microglia and astrocytes, in the ipsilateral superficial dorsal horn (Cobianchi et al., 2010, Almeida et al., 2015, López-Álvarez et al., 2015). These results suggest that inactivation of glial cells by physical exercise plays a role in producing EIH. However, our recent study showed that partial sciatic nerve ligation (PSL)-runner mice maintained a markedly increased number of microglia (microgliosis) in spite of attenuation of pain behaviors (Kami et al., 2016a). This discrepancy may be attributed to the different treadmill running protocol used in these studies, suggesting that especially the duration and intensity of treadmill running are important factors in governing analgesic levels of EIH. Also, these results suggest that attenuation of microgliosis in the spinal dorsal horn by physical exercise is not essential in producing EIH.

Recent studies have shown that pharmacological inhibition of histone deacetylases (HDACs) in the spinal cord of NPP model animals improves pain-related behaviors by reducing HDAC1 and enhancing histone acetylation (Cherng et al., 2014, Denk et al., 2013, Kukkar et al., 2014), and have also suggested that epigenetic modification plays an important role in producing and attenuating NPP (Descalzi et al., 2015). Interestingly, intrathecal administration of rat IL-10 protein or intrathecal lentiviral-mediated transfer of IL-10 can reverse the enhanced pain behaviors in CCI model rats (Cianciulli et al., 2015, He et al., 2013). Moreover, it has been shown that epigenetic modification, such as phosphorylation, acetylation and methylation of histone H3 at specific regions in the IL-10 promoter is an important regulatory step for IL-10 production in myeloid cells, including macrophages (Leng and Denkers, 2009, Zhang et al., 2006). These results suggest that epigenetic modifications in activated microglia in the spinal dorsal horn participates in producing EIH, perhaps via the up-regulation of analgesic factors, including IL-10. Our recent study showed that PSL-surgery markedly increased the number of HDAC1^+^/CD11b^+^ microglia in the ipsilateral superficial dorsal horn, while the number significantly decreased with treadmill running. Moreover, the number of microglia with nuclear expression of acetylated histone H3K9 (H3K9ace) in the ipsilateral superficial dorsal horn remained at low levels in PSL-sedentary mice, but running exercise significantly increased it (Kami et al., 2016a). Thus, our results indicate that the epigenetic modification that causes hyperacetylation of histone H3K9 in activated microglia plays a role in producing EIH. A reasonable explanation for our results may involve the up-regulation of analgesic factors, perhaps IL-10, in the activated microglia. This is the first evidence to our knowledge showing that epigenetic mechanisms are possibly involved in the EIH.

Gamma-aminobutyric acid (GABA) is the principal inhibitory transmitter in the central nervous system, including the spinal dorsal horn. GABA is synthesized from glutamate by glutamic acid decarboxylase (GAD). Two distinct isoforms of GAD, GAD65 and GAD67, have been identified, with each isoform being encoded by separate genes, namely *Gad2* and *Gad1* (Erlander et al., 1991). A line of studies indicated that the functional loss of GABA and/or GADs, especially GAD65, in the spinal dorsal horn contributes to the development of NPP via reductions in the GABA inhibitory tone (Castro-Lopes et al., 1993, Lorenzo et al., 2014, Moore et al., 2002, Vaysse et al., 2011). Our recent study showed that exacerbated pain behaviors following PSL surgery were significantly reduced by treadmill running, and PSL-induced reductions in GAD65/67 production in the superficial dorsal horn were prevented by treadmill running after the PSL surgery, leading to the retention of GABA in interneurons and neuropils. Positive correlations were also observed between the thresholds of pain behaviors and GABA and GAD65/67 levels or GABAergic interneuron numbers in the ipsilateral dorsal horn of PSL-sedentary and runner mice (Kami et al., 2016b). We further demonstrated that the reduction of GAD65, but not GAD67, is selectively prevented by exercise (Kami et al., unpublished observation). Therefore, our results demonstrated that EIH is achieved, at least in part, by the retention of GABAergic inhibition in the spinal dorsal horn. On the other hand, GADs at the protein and mRNA levels are present in the rostral ventromedial medulla (RVM), and these GABAergic RVM neurons massively project into the spinal dorsal horn (Hossaini et al., 2012, Morgan et al., 2008, Pedersen et al., 2011). These GABAergic RVM neurons were also shown to participate in pain inhibition (Zhang et al., 2011). Therefore, GABAergic neurons in the RVM may also be involved in the generation of EIH.

## The role of mesolimbic reward system in EIH

It is now clear that EIH is accomplished through multiple cellular and molecular events produced at different levels of the nervous system following physical exercise, but further studies will be required to resolve several matters. For instance, analgesic levels of EIH may be influenced by the style of exercise, i.e. forced or voluntary exercise (Sheahan et al., 2015). Our recent study showed that voluntary exercise on a running wheel attenuates pain behaviors in NPP model mice, and that its analgesic effects were equivalent to or greater than that of forced exercise such as treadmill running (Kami et al., 2016c). Moreover, it has been demonstrated that voluntary exercise is a strong natural reward (Brené et al., 2007, Werme et al., 2002), and activation of the mesolimbic reward pathway contributes to the suppression of tonic pain (Altier and Stewart, 1998, Navratilova et al., 2012). Greenwood et al. (2011) has reported that 6 week of wheel running in naive rats increased TH mRNA levels and ΔFosB/FosB immunoreactive nuclei in the ventral tegmental area (VTA) and nucleus accumbens (NAc), respectively. Therefore, it will be of interest to investigate the effects of voluntary wheel running (VWR) in NPP model animals on changes of dopaminergic neurons in the VTA, one of the key reward regions in the brain.

**Fig. 1:**
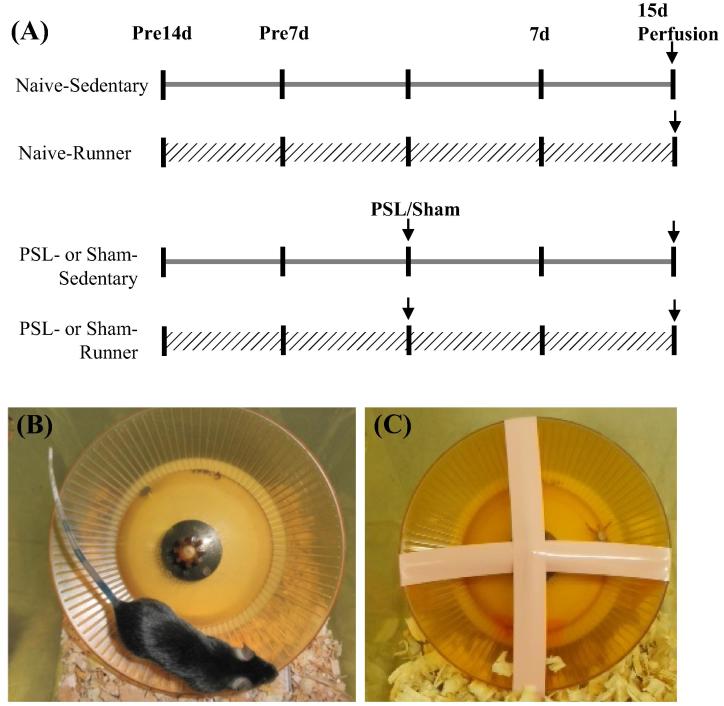
Protocols for voluntary wheel running (VWR). (A) The mice were divided into six groups: 1) Naive-Sedentary mice, 2) Naive-Runner mice, 3) Sham-Sedentary mice, 4) Sham-Runner mice, 4) PSL-Sedentary mice and 5) PSL-Runner mice. (B) Naive-, Sham, and PSL-Runner mice were allowed to run freely on the running wheel, (C) while Sedentary mice were reared in the cages with the locked running wheel. At 15 days after the surgeries, mice were transcardially perfused with 4% paraformaldehyde in 0.1 M PBS, and the brain was removed. Adapted with permission from Kami et al. (2016c).

**Fig. 2:**
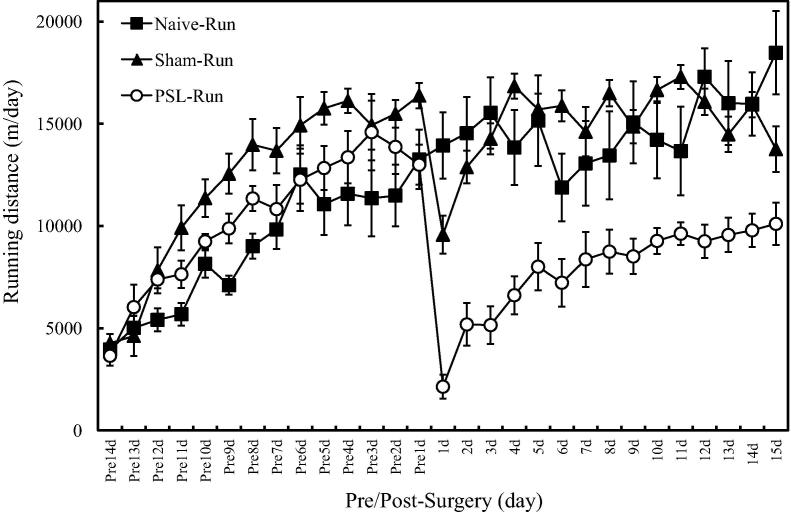
Changes of daily running distances in Naive, Sham and PSL-Runner mice throughout the experimental period. Naive-Runner (n = 5), Sham-Runner (n = 5) and PSL-Runner (n = 5) mice were placed in individual cages equipped with the low-profile wireless mouse running wheel, and daily running distances (m/day) were recorded throughout the experimental period. The distance traveled on the running wheel was monitored using a magnetic reed switch attached to a computerized exercise-monitoring system (SOF-860 wheel manager software, MED associates, Inc). Although in PSL-Runner mice, PSL surgery dramatically reduced the running distance at 1 day post-surgery, these levels gradually returned to nearly pre-surgical level at 15 days after PSL surgery. Adapted with permission from Kami et al. (2016c).

**Fig. 3:**
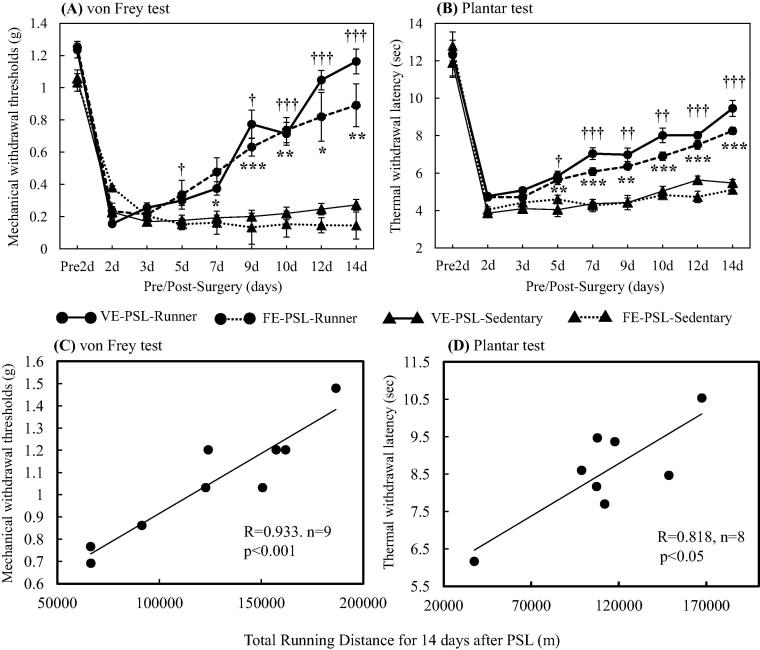
Changes of pain behaviors in mice and relationships between pain behavior thresholds and total running distances in PSL-Runner mice. (A) von Frey test and (B) plantar test were performed in VE (Voluntary exercise for 14 days)-PSL-Runner (closed circles with solid lines, n = 6), FE (Forced exercise for 14 days)-PSL-Runner (closed circles with broken lines, n = 6), VE-PSL-Sedentary (closed triangles with solid lines, n = 6) and FE-PSL-Sedentary (closed triangles with broken lines, n = 6) mice. Mechanical withdrawal thresholds and thermal withdrawal latencies were significantly higher in PSL-Runner mice compared to PSL-Sedentary mice. VE-PSL-Runner vs VE-PSL-Sedentary = †p < 0.05, ††p < 0.01, †††p < 0.001; FE-PSL-Runner vs FE-PSL-Sedentary = ^*^p < 0.05, ^**^p < 0.01, ^***^p < 0.001. Quantitative data are presented as the mean ± standard error of the mean (SEM). The significance of differences between groups was determined by Student’s *t*-test. Differences were considered significant at p < 0.05. A significant positive correlation was observed between total running distances during 15 days after PSL surgery and (C) the thresholds of von Frey (R = 0.933, p < 0.001, n = 9) or (D) the latencies of plantar tests (R = 0.818, p < 0.05, n = 8) in PSL-Runner mice. Adapted with permission from Kami et al. (2016c).

Using PSL model mice, we investigated the effects of VWR on dopaminergic neurons in the lateral region of anterior VTA (latVTA). PSL-Runner mice freely traveled on the running wheel during 15 days after PSL surgeries, while PSL-Sedentary mice were kept in the cage with the locked running wheel (Fig. 1). Although in PSL-Runner mice, PSL surgery dramatically reduced the running distance at 1 day post-surgery, these levels returned to nearly pre-surgical level at 15 days post-surgery (Fig. 2). Withdrawal thresholds of von Frey test and latencies of plantar test in PSL-Runner mice were significantly higher than those in PSL-Sedentary mice from 5 days to 15 days after the surgery (Fig.3A, B). In addition, a significant positive-correlation was observed between the assessment of pain behavioral tests and total running distances in PSL-Runner mice (Fig.3C, D).

Brain reward system, mesolimbic dopamine (DA) pathway, is composed of DA neurons in the VTA projecting to the NAc and medial prefrontal cortex (mPFC). Activation of this system is considered to cause reward and analgesia, while inactivation leads to depression and hyperalgesia. Chronic pain patients are often depressive, and pain threshold is decreased in depressive patients (Senba, 2015). Comorbidity of these two pathological states, i.e. bidirectional relationship may be due in part to shared neural mechanisms in the mesolimbic DA system. We hypothesized that EIH is induced by the activation of this DA system, since exercise did not produce EIH when DA neurons projecting from the VTA to the NAc were selectively inhibited by Gi-DREADD (designer receptors exclusively activated by designer drug) method (Wakaizumi et al., 2016).

The VTA is a heterogeneous brain structure which contains DA neurons (52%) and GABA neurons (31%) (Cohen et al., 2012), and serves a central role in motivation and reward processing. Recent studies suggest that the midbrain DA system is composed of anatomically and functionally heterogeneous DA subpopulations with different axonal projections, which may explain a number of confusing observations that suggested a role for DA in processing both rewarding as well as aversive events (for review, see Chaudhury et al., 2015). DA neurons are further divided into two parts, lateral VTA (latVTA) and medal VTA (medVTA), in which input and output projections as well as electrophysiological properties are totally different (Lammel et al., 2014).

**Fig. 4:**
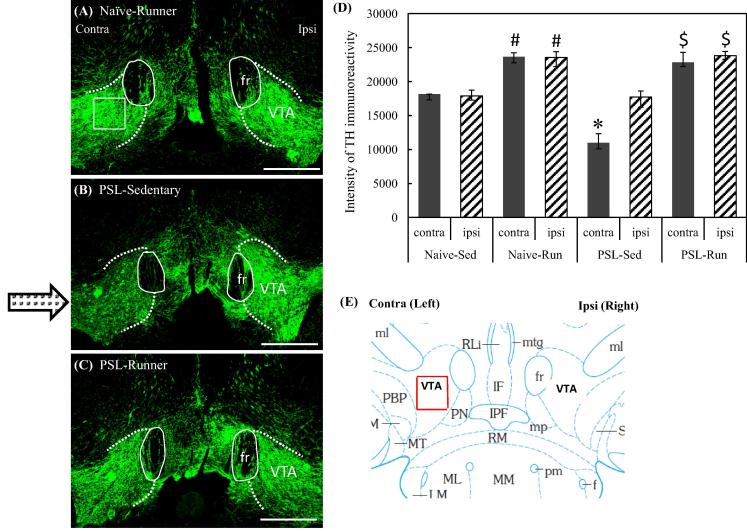
Changes of TH immunoreactivities in the latVTA by PSL with or without VWR. Brain sections (−2.92 and −3.08 mm from the bregma) in (A) Naïve-Runner (B) PSL-Sedentary and (C) PSL-Runner mice were immunostained with TH antibody. The right and left sides of the pictures indicate the ipsilateral and contralateral sides of PSL surgery, respectively. fr: fasciculus retroflexus, Bars = 300 μm. As shown in (A), a square of 200 μm x 200 μm in size was placed on the lateral region of VTA (latVTA) on microscopic images, and the immunofluorescence intensity of TH within it was quantified. (D) A bar chart showing intensities of TH-immunoreactivity in the ipsilateral and contralateral sides of latVTA in Naive and PSL mice. The intensities of TH-immunoreactivity were significantly increased by VWR (#p < 0.01 vs Naive-Sedentary, n = 5; $p < 0.01 vs PSL-Sedentary, n = 5), while the intensities of TH-immunoreactivity were significantly weaker in the contralateral side of the latVTA in PSL-Sedentary mice than those of the other groups (^*^p < 0.01, n = 5). Quantitative data are presented as the mean ± standard error of the mean (SEM). The significance of differences among groups was determined by a one-way ANOVA and Tukey-Kramer *post hoc test.* (E) Mouse brain atlas showing area in the latVTA in which the immunofluorescence intensity of TH has been analyzed (red square).

**Fig. 5:**
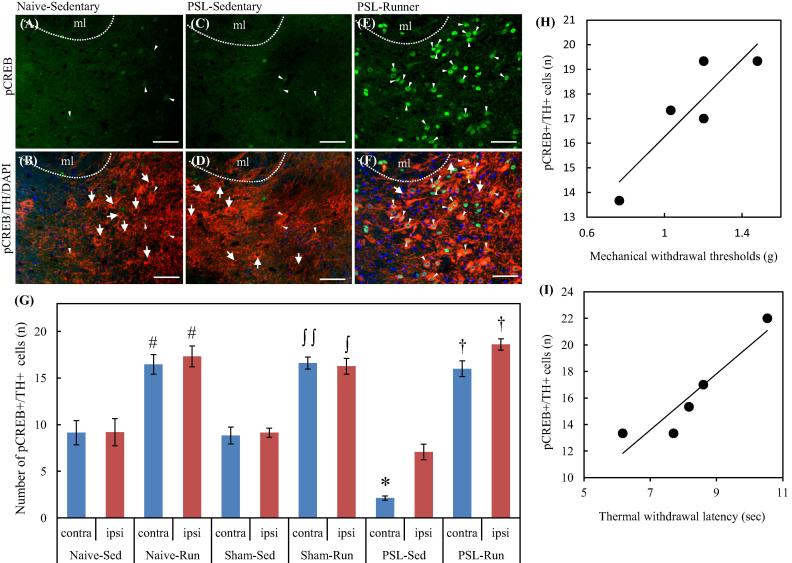
Changes of pCREB^+^/TH^+^ neurons in the lVTA by VWR. Double immunostainings with pCREB and TH antibodies were performed on the brain sections containing latVTA in each group. Photomicrographs shows localization of pCREB + cells (A, C, E) and pCREB + /TH + /DAPI + cells (B, D, F) in the contralateral side in the latVTA of Naive-Sedentary (A, B), PSL-Sedentary (C, D) and PSL-Runner mice (E, F). VWR resulted in up-regulation of pCREB in TH^+^ neurons. Arrows and arrowheads indicate TH-/pCREB + and TH + /pCREB + cells, respectively. ml: medial lemniscus. Bars = 50 μm. (G) A bar chart showing the numbers of pCREB^+^/TH^+^ cells in the latVTA of each group. The numbers of pCREB^+^/TH^+^ cells were significantly increased by VWR (# p < 0.01 vs Naive-Sedentary, n = 5; ∫∫ p < 0.01 vs the contralateral side of Sham-Sedentary, n = 5; ∫ p < 0.05 vs the ipsilateral side of Sham-Sedentary, n = 5; † p < 0.01 vs PSL-Sedentary, n = 5), while the numbers of pCREB^+^/TH^+^ cells in the contralateral side in PSL-Sedentary was significantly decreased compared with those of Naive- and Sham-Sedentary (^*^p < 0.05). Quantitative data are presented as the mean ± standard error of the mean (SEM). The significance of differences among groups was determined by a one-way ANOVA and Tukey-Kramer *post hoc test.* A significant positive correlation was observed between the number of pCREB^+^/TH^+^ neurons in the contralateral side and (H) the thresholds of von Frey (R = 0.885, p < 0.05, n = 5) or (I) the thermal withdrawal latencies of plantar tests (R = 0.932, p < 0.05, n = 5) in PSL-Runner mice.

Here we will focus on our recent findings that this system is involved in the EIH and the mechanisms how chronic pain and exercise alter the activity of this mesolimbic DA system (Kami et al., 2016c). In our immunohistochemical analysis, PSL-Sedentary mice showed a marked decrease of tyrosine hydroxylase (TH) immunoreactivities in the latVTA of the contralateral side compared with the ipsilateral side of the surgery, but VWR prevented such a decrease (Fig. 4). In addition, we tried to detect phosphorylated cyclic AMP response element-binding protein (pCREB) in TH^+^ cells, because CREB serves as a main transcriptional regulator of TH gene (Lewis-Tuffin et al., 2004). The reduced number of pCREB^+^/TH^+^ (dopaminergic) neurons in the latVTA of PSL-Sedentary mice was significantly restored by VWR in PSL-Runner mice (Fig.5G). Furthermore, we found a significant positive-correlation between the assessment of pain behavioral tests and the number of pCREB^+^/TH^+^ neurons in the latVTA in PSL-Runner mice (Fig.5H, I). In summary, VWR increases the expression of pCREB in the dopaminergic neurons in the latVTA of PSL mice (Fig. 5), which would enhance DA production, and thereby contributes to the activation of the mesolimbic reward system in PSL-Runner mice. Therefore, we conclude that EIH may be achieved, at least in part, by activation of the mesolimbic reward pathway. We are now investigating how exercise can activate VTA neurons.

**Fig. 6:**
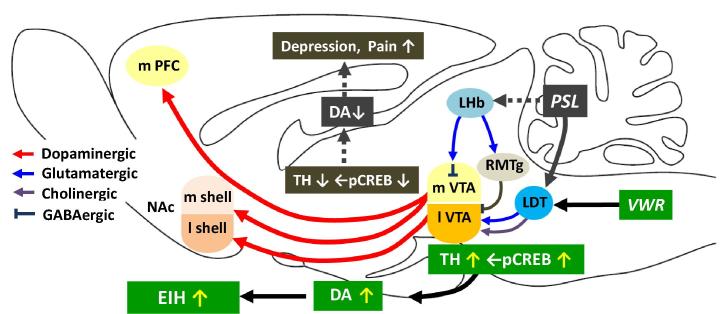
Simplified schematic drawing of the major neural circuit connections involved in EIH. The network displays the complex interplay in regulating cellular activity within the reward system and several nuclei projecting to the VTA. GABA: gamma-aminobutyric acid, LDT: laterodorsal tegmental nucleus, LHb: lateral habenula, mPFC: medial prefrontal cortex, NAc: nucleus accumbens, PPTg: pedunculopontine tegmental nucleus, RMTg: rostromedial tegmental nucleus, VTA: ventral tegmental area.

Input-specific control of reward and aversion in the VTA has been demonstrated by Lammel et al. (2012). There are two main input sources to the VTA, one is the lateral habenular nucleus (LHb) and the other is the pontine tegmental nuclei, i.e. laterodorsal tegmental nucleus (LDT) and pedunculopontine tegmental nucleus (PPTg). Chronic pain or other aversive stimuli activates Glu-containing LHb neurons, which then project to GABA neurons in the VTA and more caudally located rostromedial tegmental nucleus (RMTg) to inhibit DA neurons in the VTA. Thus chronic pain decreases the VTA-NAc DAnergic projection and produce aversive behavior (Matsumoto and Hikosaka, 2007). On the other hand, reward stimuli activate LDT and PPTg neurons, which then activate DA neurons in the VTA and reward system, causing preference behavior (Fig. 6).

**Fig. 7:**
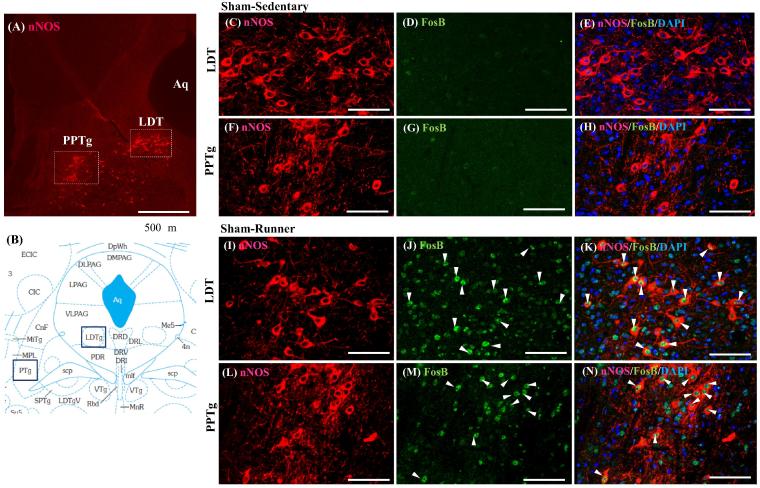
Voluntary exercise increases ΔFosB/FosB expression in the LDT/PPTg neurons. Double immunostaining with nNOS and ΔFosB/FosB antibodies was performed on the brain stem sections containing LDT/PPTg. The locations of the LDT and PPTg containing nNOS + (cholinergic) neurons in the midbrain are indicated as squares in (A). Aq: aqueduct. (B) Mouse brain atlas showing areas of LDT/PPTg (blue squares). No ΔFosB/FosB is expressed in LDT (C, D, E)/PPTg (F, G, H) neurons in Sham-Sedentary mice, while numerous ΔFosB/FosB positive nuclei were observed in both nNOS + (arrowheads) and nNOS- neurons in the LDT (I, J, K)/PPTg (L, M, N) in Sham-Runner mice. Bars = 50 μm.

It is reasonable to assume that exercise-induced excitatory input to the VTA may come from the LDT/PPTg, which receives inputs from cerebral cortices, including PFC and motor area, and basal ganglia, and then provides strong excitatory inputs (glutamatergic and cholinergic) to DA neurons in the basal ganglia and VTA (Mena-Segovia et al., 2008). It has been demonstrated in rats that about a third of LDT/PPTg neurons are cholinergic and the rests are glutamatergic and GABAergic with a minimal overlap (Wang and Morales, 2009). Then we examined if these neurons are activated by voluntary running using ΔFosB/FosB as a marker of neural excitation. In runner mice, a considerable number of LDT and PPTg neurons, both nNOS^+^- cholinergic and non-cholinergic (putative glutamatergic) neurons exhibited ΔFosB/FosB expression (Fig. 7), indicating that LDT/PPTg-VTA pathway may be activated by exercise. Some of them are also activated by PSL surgery, since nocifensive on- and off-cells are identified in the LDT/PPTg (Carlson et al., 2005).

## Exercise-induced changes in functional connectivity in the brain

A recent remarkable advance in neuroimaging methods has enabled a visualization of ongoing clinical pain. One such method is functional connectivity magnetic resonance imaging (fcMRI), a noninvasive technique applied in wakeful humans either at rest (i.e., resting-state connectivity) or during task. With resting-state analyses, low frequency (<1 Hz) temporal fluctuations in the MRI signal are assessed across various brain regions, which are thought to indicate functional connectivity between brain regions.

Central sensitization and descending facilitation may play important roles in the pathogenesis and maintenance of pain in functional pain syndrome or central dysfunctional pain such as fibromyalgia (FM), chronic low back pain (CBP) and temporomandibular disorder (TMD) (Senba et al., 2011). Characteristic functional connectivity of brain networks in chronic pain patients has been reported. For example, increased insular connectivity to the default mode network (DMN), a network whose activity is increased during non-task resting states, was also observed in FM, TMD or CRPS patients (Bolwerk et al., 2013, Ichesco et al., 2014, Kucyi et al., 2014). Following 4 weeks of nonpharmacological interventions, such as acupuncture or cognitive behavioral therapy, intrinsic DMN connectivity to the insula was reduced, and this reduction correlated with reductions in pain (Napadow et al., 2012). Moreover, aberrant resting state functional connectivity of insula in FM patients was normalized following three months of physical exercise therapy (Flodin et al., 2015). Activity in the insula, an area related to pain unpleasantness, intensity coding, anxiety and depression, is augmented in FM patients compared to controls in response to painful stimuli, and this augmentation was negatively correlated to physical activity (Ellingson et al., 2012). On the other hand, negative relationships between sustained sedentary time and brain activity in the dorsolateral prefrontal cortex (DLPFC) (Ellingson et al., 2012), which has been shown to be involved in the top-down modulation of pain (Lorenz et al., 2003). As FM patients tend to be highly sedentary (McLoughlin et al., 2011), even a low level of physical activity may be able to modulate their pain. These findings suggest that exercise training is a consistently beneficial treatment for chronic pain patients.

Fear of movement (FOM) has been increasingly recognized as a significant explanatory factor for developing chronic pain (chronification of pain). According to the Fear-avoidance model (Lethem et al., 1983), the development of FOM is characterized by a vicious circle of various cognitive and behavioral aspects such as pain catastrophizing and avoidance behavior that may ultimately lead to physical deconditioning of the musculoskeletal system (Vlaeyen and Linton, 2000). A recent fcMRI study in CBP patients has demonstrated differential effects of fear of movement between patients with CBP and pain-free subjects were found in the extended amygdala and in its connectivity to the anterior insula (Meier et al., 2016), which may provide a neurobiological basis for the Fear-avoidance model. Exercise and elevating the level of spontaneous activity in daily life by overcoming fear and avoidance behaviors may be a key to solve the vicious circle of the Fear-avoidance model.

## Factors that determine physical activity/physical inactivity

Sedentary behavior, sitting inactive for long period of a day, will lead to various lifestyle-related diseases, such as type2 diabetes, hypertension, cerebrovascular disease and ischemic heart disease (Hamilton et al., 2014). How to increase our ADL is the critically pressing matter of our society. Although it is well established that regular exercise is a health benefit for all individuals, a meta-analysis study (Rhodes and de Bruijn, 2013) showed that 36% of individuals had the intention to exercise but failed to implement the intention, and 21% never had any intention to exercise, which seems to be much worse than mice.

In our VWR experiments, about 10% of mice did not want to run at all in 2 weeks of pre-surgery period and they were omitted for further experiment. In human, some people are active and others are inactive. Then a question arises how physical activity (PA)/ inactivity (PI) is determined. This disposition seems to be differentially encoded in our neural system genetically and/or epigenetically. It has been demonstrated in animals that increased levels of DA, orexin, histamine etc. in the brain may contribute to hyperactivity (Viggiano, 2008), while lesions of mesolimbic DA system produce hypoactivity (Koob et al., 1981), strongly suggesting the important roles of this system in the regulation of physical activity. While many genetic and neural PA/PI factors are reported for animals, human studies are relatively few. In one meta-analysis study of 45 reports published from 1980 to 2010, candidate genes that determines human activity/inactivity were postulated, in which significant associations with PA phenotype were found for *ACE (angiotensin I-converting enzyme), Gln223Arg*, *MC4R (melanocortin 4 receptor)*, *DRD2 (D2 dopamine receptor)* genes (de Vilhena et al., 2012) . An association study of Loos et al. (2005) reported a significant interaction between MC4R-C-2745T and PA levels. T/T homozygote offspring showed higher inactivity scores than heterozygotes or C/C genotype. Two studies performed in mice were able to identify *NHLH2 (nescient helix-loop-helix 2)* as able to promote motivation for exercise (Good et al., 2008) and *GLUT4* as capable to alter PA levels through increased glucose influx (Tsao et al., 2001). Two SNPs in the human *NHLH2* gene, which affects the function of NHLH2 protein, were recently identified (Al Rayyan et al., 2013).

**Fig. 8:**
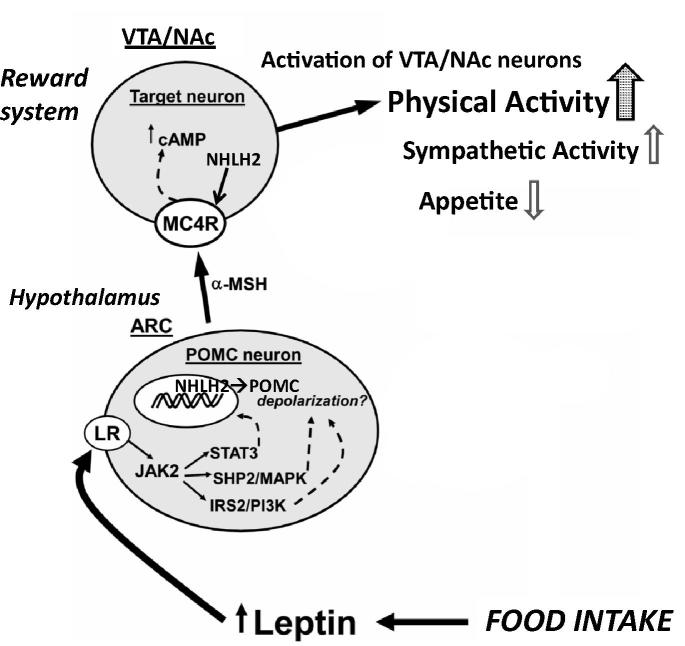
Schematic representation of the interaction between hypothalamic POMC neurons and the reward system. Although this schematic representation highlights the importance of POMC neurons located in the ARC and projecting to MC4R containing second-order neurons in the paraventricular nucleus and lateral hypothalamic area to control appetite and energy metabolism, they also project to MC4R containing neurons in the reward system, such as VTA and NAc, to positively control physical activity. Thus, hypothalamus and reward system control energy metabolism in a concerted manner. Synthesis of MC4R is under the control of a transcription factor NHLH2, a key molecule that control physical activity and fitness. ARC: arcuate nucleus of the hypothalamus, cAMP: cyclic adenosine monophosphate, LR: leptin receptor; MC4R: melanocortin 4 receptor; POMC: proopiomelanocortin protein; α-MSH: α-melanocyte stimulating hormone; Modified from Fig. 2 of da Silva et al. (2013).

Here, we focus on *NHLH2* and *MC4R* genes among these candidate genes. For example, *NHLH2* KO mice show physical inactivity in young age (5–8 weeks) and overeating and obesity after 12 weeks, which resembles human middle age obesity (Coyle et al., 2002) . One of the target genes of NHLH2 is MC4R (Wankhade and Good, 2011), a receptor for α–melanocyte stimulating hormone (MSH), one of proopiomelanocortin (POMC) gene products. POMC neurons in the arcuate nucleus (ARC) in the hypothalamus produce α–MSH, which activate second-order hypothalamic neurons expressing MC4R to inhibit appetite and activate energy metabolism. These neurons also project to the reward system, in which VTA DA neurons and NAc neurons express MC4R (Pandit et al., 2013) (Fig. 8). Activation of these neurons may lead to an increase of physical activity, contributing to the energy balance and physical fitness. On the other hand, various mutations in the MC4R gene are implicated in 1–6% of early onset or severe adult obesity cases in humans (Lubrano-Berthelier et al., 2003).

Thus, the hypothalamus and reward system is closely related in the axis of food intake, energy metabolism and physical activity. Understanding the interactions between the mesolimbic DA system which mediates the incentive salience of natural and artificial rewards and the neural and hormonal systems that sense and regulate energy balance is thus of significant importance. Therefore, in a certain sense, chronic pain and obesity may share common behavioral and neural pathology, i.e. physical inactivity, as a result of inactivation or dysfunction of the mesolimbic DA system. Recently, Janke et al. (2016) reported that 24% of respondents of their survey reported persistent pain, and had significantly higher BMIs than their pain-free peers. They applied the Fear-avoidance model of pain to explain the relationship between pain and increased weight; obesity contributes to chronic musculoskeletal pain, impairment of mobility, and eventual physical disability and psychosocial characteristics of chronic pain, i.e. pain catastrophizing, kinesiophobia, and depression.

Another target gene of NHLH2 is monoamine oxidase-A (MAO-A), which is considered to be related to sex difference of physical activity since it is encoded in X-chromosome (Good and Li, 2015). The transcriptional activity of NHLH2 is determined by the number of VNTR (variable number tandem repeats) site in the promoter region of MAO-A gene. Higher translation and activity of MAO-A may lead to higher metabolism of monoamines, such as DA and 5-HT. When the level of DA and/or 5-HT is low, the pain threshold will be lowered in some women, and they become more vulnerable to fear and anxiety, easily getting catastrophic and as a consequence they might suffer depression, panic disorder or chronic pain. This may be one of the reasons why chronic pain, such as FM, TMD is more prevalent among women than men.

## Conclusion

Thus, physical exercise has been established as a low-cost, safe, and effective way to manage chronic intractable pain. It can reduce the dose of drugs used and eventually their side effects. It will improve the quality of life and give us a power of living by activating brain reward system. Chronic pain could be recognized as a lifestyle-related disease, since chronic pain is at least partly attributed to sedentary and inactive lifestyle as indicated by the Fear-avoidance model, which will necessitate our serious effort to change our lifestyle, inactive to active.

## Conflict of interest

The authors declare that they have no conflicts of interest.
